# Silent EEG-Speech Recognition Using Convolutional and Recurrent Neural Network with 85% Accuracy of 9 Words Classification

**DOI:** 10.3390/s21206744

**Published:** 2021-10-11

**Authors:** Darya Vorontsova, Ivan Menshikov, Aleksandr Zubov, Kirill Orlov, Peter Rikunov, Ekaterina Zvereva, Lev Flitman, Anton Lanikin, Anna Sokolova, Sergey Markov, Alexandra Bernadotte

**Affiliations:** 1 Experimental ML Systems Subdivision, SberDevices Department, PJSC Sberbank, 121165 Moscow, Russia; DVVorontsova@sberbank.ru (D.V.); AIZubov@sberbank.ru (A.Z.); bernalis@yandex.ru (P.R.); esezvereva@sberbank.ru (E.Z.); leflitman@sberbank.ru (L.F.); ABLanikin@sberbank.ru (A.L.); AAlekSokolova@sberbank.ru (A.S.); markov.s.s@sberbank.ru (S.M.); 2 Software Engineering Department, National Research University of Electronic Technology (MIET), 124498 Moscow, Russia; 3 Faculty of Mechanics and Mathematics, Moscow State University, GSP-1, 1 Leninskiye Gory, Main Building, 119991 Moscow, Russia; menshivan@phystech.edu; 4 Department of Control and Applied Mathematics, Moscow Institute of Physics and Technology (MIPT), 141700 Dolgoprudny, Russia; 5 Department of Information Technologies and Computer Sciences, National University of Science and Technology MISIS (NUST MISIS), 119049 Moscow, Russia; 6 Research Center of Endovascular Neurosurgery, Federal State Budgetary Institution “Federal Center of Brain Research and Neurotechnologies” of the Federal Medical Biological Agency, Ostrovityanova Street, 1, p. 10, 117997 Moscow, Russia; rensorlov@icloud.com; 7 Russia Endovascular Neuro Society (RENS), 107078 Moscow, Russia

**Keywords:** brain–computer interface, neurorehabilitation, neurodegeneration, neurodegeneration treatment, senescence, eSports, deep learning, imagined speech, silent speech, speech recognition, EEG, EEG-BCI

## Abstract

In this work, we focus on silent speech recognition in electroencephalography (EEG) data of healthy individuals to advance brain–computer interface (BCI) development to include people with neurodegeneration and movement and communication difficulties in society. Our dataset was recorded from 270 healthy subjects during silent speech of eight different Russia words (commands): ‘forward’, ‘backward’, ‘up’, ‘down’, ‘help’, ‘take’, ‘stop’, and ‘release’, and one pseudoword. We began by demonstrating that silent word distributions can be very close statistically and that there are words describing directed movements that share similar patterns of brain activity. However, after training one individual, we achieved 85% accuracy performing 9 words (including pseudoword) classification and 88% accuracy on binary classification on average. We show that a smaller dataset collected on one participant allows for building a more accurate classifier for a given subject than a larger dataset collected on a group of people. At the same time, we show that the learning outcomes on a limited sample of EEG-data are transferable to the general population. Thus, we demonstrate the possibility of using selected command-words to create an EEG-based input device for people on whom the neural network classifier has not been trained, which is particularly important for people with disabilities.

## 1. Introduction

Some neurological conditions and diseases are accompanied by a loss of communication and movement functions. Such diseases include, for example, amyotrophic lateral sclerosis, Parkinson’s disease, multiple sclerosis, various infectious diseases, stroke, injuries of the central nervous system, locked-in person syndrome, hereditary and acquired polyneuropathies, developmental disorders, and cancer. Recent data show serious neurological consequences after COVID-19. Due to the aging population in developed and developing countries and the epidemic of infectious diseases with neurological consequences, there is a growing need for the rehabilitation and adaptation of patients with neurological disorders. In the modern world, there is a trend towards inclusiveness, which inspires us, as a society, to explore new technologies to accommodate people who are excluded from normal interaction.

In our work, we investigated the possibility of developing a brain–computer interface (BCI) with a manipulator for people with disabilities by decoding the mental commands: ‘forward’, ‘backward’, ‘up’, ‘down’, ‘help’, ‘take’, ‘stop’, and ‘release’. Taking into account the specific reasons behind patients’ loss of movement and communication functions, we considered the possibility of finding patterns among commands thoughts in the population, in order to be able to use BCI trained on healthy people to help people with disabilities in the future.

Due to technological and biotech progress, the field of BCI is actively developing at the moment. A BCI is a device which provides communication between the brain and artificial devices via converting analog brain signals into digital signals, which are recognizable by machines. Devices that recognize brain activity can be invasive and non-invasive. Invasive BCIs can be intracortical, with electrodes located inside the brain, and supracortical—electrocorticography-based BCIs [1,2]. The size of the devices and electrode material used is selected in order to minimize direct and secondary brain damage (a local inflammation reaction).

The vast majority of invasive BCIs collect information from a strictly localized brain area, for example, the motor cortex. Most invasive devices work via the recognition of basic motor skills such as high-amplitude hand movements. However, there is research and development aimed at reading more subtle and specific information. In 2021, Stanford University researchers demonstrated an invasive BCI implanted in the motor cortex that can decode the imaginary micromovements of a paralyzed person’s hand and translate them into text in real-time. As text input devices, the authors surpassed other BCIs in accuracy and speed and reached a speed of 90 characters per minute with an accuracy of 94.5–99% [3]. Despite the significant success of invasive devices, it should be understood that invasive BCIs carry a high risk of local nonspecific brain inflammation and infection, especially for immunocompromised patients. Consequently, these devices can be applied to a narrow group of individuals, and in this vein, non-invasive BCIs are of much greater interest.

Non-invasive BCIs, such as EEG-based BCIs, can be applied much more extensively than invasive ones. Thus, non-invasive devices do not damage the brain, do not require major surgery, are easy to maintain, and are available to many people in need. EEG-based BCIs record electrical potentials from the surface of the head that arise during brain activity. Thus, the EEG reflects the functional activity of the brain, obtained as a projection onto the surface of the head at discrete moments in time.

In common practice, EEG-based BCIs have from 8 to 256 or even more channels that allow simultaneous recording of electrical activity from a corresponding number of electrodes installed on the surface of the head. In an EEG-based BCI, one channel records the total electrical activity of many millions of neurons, represented mainly by the potentials of the dendrites and bodies of the neurons of the cortex, projected onto a certain area of the head surface. Moreover, only a synchronized signal from a large number of neurons reaches the surface of the head. In this case, the activity of individual small sections of neurons, morphologically or functionally similar, will not be recorded [4].

This feature of an EEG-based BCI to obtain the data projected onto the surface of the head gives us an understanding of the boundaries of the capabilities of an EEG-based BCI. A blurry signal from many neurons in the brain, combined with the superposition of external technical noise, is very difficult to recognize and interpret. However, it is also possible to build a device that recognizes patterns from such data.

To demonstrate the capabilities of non-invasive EEG-based BCIs in the study of decoding a brain signal when subjects are watching a video, let us turn to the work of researchers from the Moscow Institute of Physics and Technology. The authors of this paper learned to identify specific patterns of brain activity for five classes of images (in the form of a video sequence) and successfully classify these five groups of images, receiving an electrical signal of brain activity from 256 non-invasive electrodes [5]. Earlier, in 1994, interesting results were obtained using an elegant solution for parallel training of a Siamese neural network (two identical networks with common weights) on the data of the displayed visual series and the EEG recorded at that moment [6].

Recognizing images based on the electrical activity of the brain is a difficult task because we do not have a clear understanding of how the projective and associative cortex centers of the brain process visual information and what kind of patterns will hold constant across the population. However, the visual cortex is a rather conservative structure of the brain, an evolutionarily ancient structure, so we can expect to find patterns of brain activity that are invariable in the population. A completely different situation arises when we look at the speech center of the human brain. The speech center can vary greatly among different language groups and people with different levels of language proficiency. To analyze the electrical signal from the speech center, it makes sense to remove the signal from the motor cortex, which is activated when pronouncing words. The signal from the motor cortex can be removed by using silent speech. While recognition of movements by EEG is already a fairly simple task in terms of recognizing patterns of movement on a recording of electrical activity of the brain, speech without involvement of the motor cortex or “silent speech” recognition is a real challenge. Silent speech recognition, besides being very difficult, is very important for people with motor limitations, paralyzed people, and people with locked-in syndrome. Thus, the field of EEG-based BCIs that recognize silent speech, or rather, recognize a strictly fixed dictionary is interesting and promising. The recognition of silent commands from a fixed dictionary is the first step in the field of silent speech recognition. The command words have a semantic component and applied meaning for creating silent speech based BCIs.

The first works on the recognition of silent speech from EEG-data were focused on statistical features and the nature of the distribution of the received signals. In 2009, Charles DaSalla and his colleagues proposed an algorithm for recognizing English phonemes /a/ and /u/ [7]. Another related study of imaginary phonemes was conducted in 2010 at a conference on bioinformatics and biomedical engineering at Carnegie University, where an algorithm based on the use of autoregressive coefficients as features and the k-nearest neighbors algorithm as a classifier of two imaginary syllables /ba/ and /ku/ was presented [8]. Later, the recognition of phonemes from EEG-data became quite popular among neurophysiologists and device developers. In 2016, Korean researchers used statistical features such as mean, variance, standard deviation, and asymmetry for the paired classifications of the vowels /a/, /e/, /i/, /o/, and /u/. As a result, the authors came to the conclusion that an extreme learning machine with a radial basis function and the use of linear discriminant analysis gives higher accuracy (the average accuracy of this model was 72%) [9,10].

Of course, researchers and BCI developers are eager to be able to recognize words, not phonemes, because this would allow developers to use BCIs as input devices without developing a special vocal vocabulary. The difficulty of recognizing mental words caused researchers to select words that, as they thought, had little semantic or phonetic similarity. In 2017, researchers from the Birla Institute of Technology and Science (India) presented a method for recognizing two English (‘yes’, ‘no’) and two Hindi (‘haan’, ‘na’ ) words. The researchers used a neural network to classify such low-dimensional data. As a result, an accuracy of 75.4% was obtained on paired combinations of English and Hindi words [11].

In 2017, colleagues from Arizona State University published a method based on the use of covariance matrix descriptors lying in the Riemannian manifold, considered as features, and the method of corresponding vectors as a classifier. As a result, when recognizing English vowels /a/, /i/, and /u/, an accuracy of 49.2% was obtained; for the words ‘in’ and ‘out’ the accuracy was 50.1%; for long words such as ‘cooperate’ and ‘independent’ it was 66.2%; and the accuracy reached 80.1% when recognizing the short word ‘in’ and the long word ‘cooperate’ [12]. In 2018, at a conference in the UK, an algorithm for recognizing 11 phonemes and 4 English words with an accuracy of 33.33% was published. A mel-frequency cepstrum (MFC) was used and the obtained data was classified using a support vector machine (SVM) [13].

In 2019, scientists from India published a method for recognizing seven syllables, /iy/, /piy/, /tiy/, /diy/, /uw/, /m/, and /n/, and four words, pat, pot, knew, and gnaw, on data from 11 EEG channels located in areas of the cerebral cortex involved in speech presentation [14]. Due to the high correlation between different channels, each channel was used as a separate object for the neural network training. For preliminary data processing, the authors divided the EEG signals into the 7 levels using a wavelet transform, captured specific frequency bands that had information about cortical activity corresponding to silent speech, and trained a neural network with two hidden layers. According to the authors, the classification accuracy reached 57.15% on average, which is a very high result, taking into account the modest dataset in terms of volume and the modest number of parameters of the neural network.

In 2019, researchers from the laboratories of the University of British Columbia presented work on silent speech recognition using hierarchical deep learning [15]. The first level of the hierarchy was used to extract spatial features from the covariance matrix and consisted of a convolutional neural network with two convolutions and fully connected layers. To study the hidden temporal features of EEG-data, a 6-layer convolutional neural network was used in parallel on the channel covariance matrix [16]. The results of the last fully connected layers of these networks were combined into a single feature vector and fed to the input of the second level of the hierarchy—a deep autoencoder [17]. The autoencoder contains three coding and three decoding layers and its output was transferred to the classification layer of extreme gradient boosting, which gave the final prediction for the phonological classes of imaginary speech [18]. As a result of training the presented hierarchical model, the authors received an accuracy of 83.42% for 6 phonemes binary classifications and 53.36% for words binary classifications. Compared to previous work, such results seem convincing and are consistent with those of other scientists.

In 2020, researchers with the same surname (Dong-Yeon Lee and Minji Lee and Seong-Whan Lee) published a classification algorithm that uses both supervised and unsupervised learning approaches. The work used the SimenseNN network. Two randomly selected EEG samples were fed to the input of SimenseNN, each of the samples trained its own CNN branch. If both samples belong to the same class, the common (embedding) layer selects such parameters so that these samples were close, otherwise it increased the distance between them. As a result, SimenseNN found the distances between words in a spatial space [19,20]. Later, researchers from Canada published an algorithm with feature extraction using a wavelet transform and a multilayer regularized neural network to classify the imaginary choice between ’yes’ and ’no’ with 67% accuracy [21].

In 2018, scientists from the MIT Research Laboratory introduced AlterEgo, a personalized wearable silent speech BCI [22]. The authors claimed to have been able to classify silent speech using a three-layer network with an accuracy of 92.01%. Unfortunately, the scientists refused to provide the assembled dataset for testing, and the modesty of the neural network architecture they used to classify the silent speech raises many questions. There is reason to believe that the dataset was for the most part assembled on one individual and during the work the individual was getting used to the existing neural network classifier. In addition, it should be noted that this BCI did not use EEG, but myography, since the electrodes were located on the face and neck, in the area of facial nerve abduction, making it able to record the muscle signals that arose during silent speech.

Taking into account the presented works, the average recognition accuracy for the binary classification of words or syllables approached 72% with a maximum accuracy of 85.57% [7,8,9,11,14,15,21,23,24]. For four words, according to the papers presented, the classification accuracy averaged 59%, while the maximum accuracy reached 99.38% [13,15,23,25]. The data of these works are presented in Table 1.

A prerequisite for the development of EEG-based BCIs is the availability of data representing electrical activity in the brain, sufficient for a particular task, collected on a device with similar technical characteristics as the one being developed. Currently, many development and research teams are sharing the EEG-data they have gathered in their work on BCI-related projects. Despite the significant advantage of this phenomenon, open datasets have some drawbacks, which, to be fair, are also found in closed datasets.

First, there is no single standard for collecting data. This leads to poor compatibility of data from different datasets and the complexity of training networks on heterogeneous data. BCI devices have a different number of channels, different signal quality, and different physical properties of the recording device. In addition, data are stored in different formats that require separate preprocessing.

Secondly, there is no internal quality control of the data in the dataset. This point is related to the peculiarity of brain activity data itself, the marking of which always implies some unreliability. For example, when collecting EEG-data while recording silent speech, it is difficult to determine from the data whether the subjects were speaking with an inner voice, how they were focusing, and so on.

Third, it is difficult to select criteria for falsifying data, which leads to training neural networks on poorly labeled data. For example, in the well-known and commonly used KaraOne dataset, we found that 6 out of 12 records are actually one biased record (whereas, according to the authors of the dataset, each record corresponds to one subject).

All of these shortcomings lead to the fact that there are not that many compatible datasets for training classifiers to recognize certain patterns for specific tasks. One of the ways to solve the problem of lack of data is to create open data aggregators with a single standard. The initiative to collect open datasets has been gaining momentum over the past 2 years. One popular open data resource with good potential for EEG-based BCI is the OpenNeuro project [29].

EEG-data widely used for speech recognition falls into two broad groups: data for sound EEG-pattern recognition and for semantic EEG-pattern recognition [30]. The first group’s paradigm is based on the hypothesis that sound itself is an entity, represented by various excitations in the brain. Indeed, adding a strong signal such as EMG data or vibration to assist the EEG-data can help to classify sounds. The second group’s paradigm is that the meaning of a word has a serious value in what kind of electrical signal can be generated in the brain.

Another orthogonal division of data that we can find in articles on this topic is the separation of tasks into word classification and speech recognition in EEG-data. However, it must be admitted that scientists dream of being able to recognize speech from EEG-data as freely as it is now performed on ordinary speech [31].

Two more features of the existing datasets should be mentioned. First, most EEG-datasets are based on English vocabulary. Thus, the representation of Slavic and other languages is very modest [32]. Second, the total set of the words itself is rather thin. Usually researchers do not go beyond a couple of dozen words. Expanding the size of the dataset is an urgent problem, since the size of the dataset directly depends on how deep neural networks we can be used to classify speech.

In our study, we studied the recognition of unspoken speech using EEG-data. The choice of silent speech for our research was characterized by two reasons.

- The first is the main applied goal. Our group is developing a BCI for people with communication difficulties, locked-in syndrome and other serious difficulties in articulation. Thus, we have to focus on minimizing the contribution of verbal communication in our data.
- The second is convenience of signal processing in less noisy data. Using only silent speech we had less distortion from motor center activation obscuring our speech center data. When working with an EEG, articulation itself and other small movements such as blinking and head movements cause serious noises. In our dataset, we tried to reduce the contribution of motion to our data, assuming that we can add this kind of noise later.

Despite the fact that we have tried to reduce motion noise, collecting a dataset is a difficult task without explicit criteria for its quality. Thus, subjects who misunderstood the task can introduce significant distortions into the dataset. The absence of an easily formalized criterion for data quality when working with silent speech requires more careful work with additional dataset criteria: understanding the type of data distribution, distribution parameters, the presence or absence of clusters in the data, highlighting outliers, and detecting a systematic error. In our work, we assumed that our data may contain clusters determined by both unchanging and temporary states in the subjects (for example, during sleepiness, fatigue, or anxiety).

In addition, we wanted to understand how consistently and uniformly the individual performs silent speech tasks. We wanted to answer the question of how strong individual ontogenesis is; that is, whether people differ in their development so much that it makes sense to train the neural network classifier on an individual user when developing BCIs. Of course, we wanted to answer the question, in general, does it make sense to train a neural network on a large silent speech dataset? Are there any constants in the brain activity of silent speech? Can we expect that increasing the dataset will increase the accuracy of silent speech recognition? This is how we formulated our hypotheses.

**Hypothesis** **1.** *Individual brain development has patterns of electrical activity of the brain that is constant across a single language community when solving silent speech problems. The learning outcomes on a limited sample are portable to the general population.*

That is, this hypothesis asserts that the human population of one language group has some patterns of brain activity that would allow us, upon their activation on the EEG, to see what word the individual is silently pronouncing or thinking. Of course, we would like to formulate and test a hypothesis regarding several language groups in order to understand how strongly the same semantic words differ in EEGs in different language groups. However, in this article we present the results shown for Slavic speakers.

While expecting to see patterns of electrical activity across the entire population, we wanted to evaluate the contribution of individual brain development to the formation of individual patterns during silent speech. Thus, hoping to answer the question whether it is better to train a silent speech neural network classifier on one individual or several, we formulated the following hypothesis.

**Hypothesis** **2.** *A smaller dataset collected on one subject makes it possible to build a more accurate classifier for a given subject than a larger dataset collected on a group of people.*

In addition to the homogeneity of the population with respect to silent speech, we were interested to know how strongly the words are similar to each other with respect to the activity they elicit in the brain during silent speech.

**Hypothesis** **3.** *There are words that share similar patterns of brain activity.*

In addition, we were interested in how we can better conceptualize EEG-data. The EEG signal is not quite a sound and not quite a picture. On the one hand, our data can be viewed as multichannel sound, where each “sound” channel is data from a corresponding EEG electrode. At the same time, the data sampling rate is very low for using classical neural network models for speech recognition. This means that we could not use classical neural networks that would accept data as input in the form of, for example, a mel-spectrogram. On the other hand, our EEG-data can be viewed as a sequence of pictures that change over time. When preparing the data and training the network, we used both paradigms. In addition, we paid special attention to data preprocessing, trying to “help” the neural network find features in the data.

## 2. Materials and Methods

### 2.1. Collecting the EEG Data

All subjects had reached adulthood, were healthy, and signed voluntary consent to the study. The subjects could interrupt the study at any time without explanation. The subjects provided their data, which included: gender, age, education, and occupation. The exclusionary criteria for the study were a history of head trauma, alcohol or other intoxication, and epilepsy. There were 268 healthy subjects in total.

**Figure 1:**
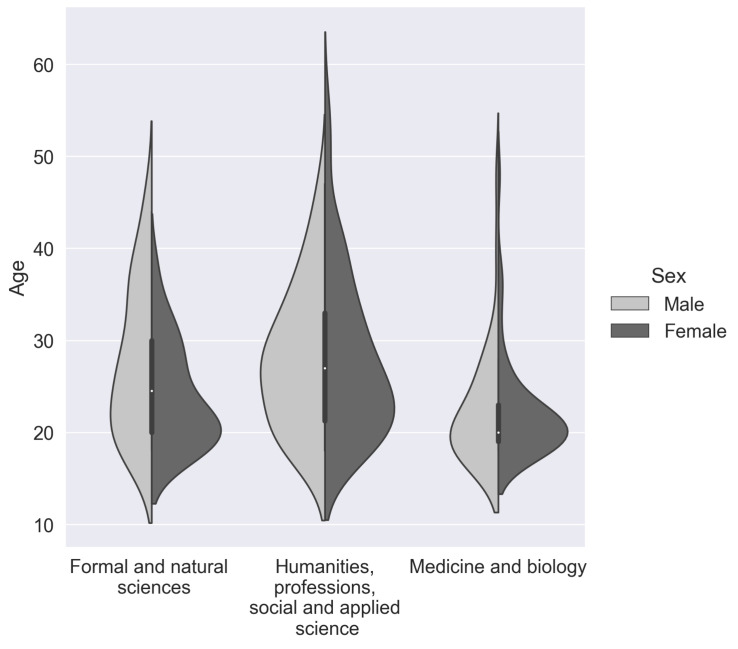
Subjects, grouped by age, education (bachelor degree or higher—left plot, undergraduate and secondary special education—right plot), gender, and professional area of current work.

We grouped our data into three categories according to the participants field of occupation. In the “Formal and natural sciences” category, we combined technical specialties, including mathematics, physics, computer science, and engineering. The “Formal and natural sciences” group consisted of 27 females and 39 males. Subjects engaged in biology or studying medicine and biology were separated into a single cluster—“Medicine and biology”. The “Medicine and biology” group consisted of 63 females and 33 males. A very heterogeneous group called “Humanities, general workers, social and applied science”, which included people of the humanitarian area and 2 general workers, consisted of 63 females and 33 males. Our subjects’ social data can be found in more detail in Figure 1 and Table 2.

Our dataset was recorded from healthy subjects during silent and vocalized speech of 8 different Russia words (commands): ‘forward’, ‘backward’, ‘up’, ‘down’, ‘help’, ‘take’, ‘stop’, and ‘release’. As a control, we also produced pseudowords, which were sequences of 4 to 6 pseudoletters.

**Figure 2:**
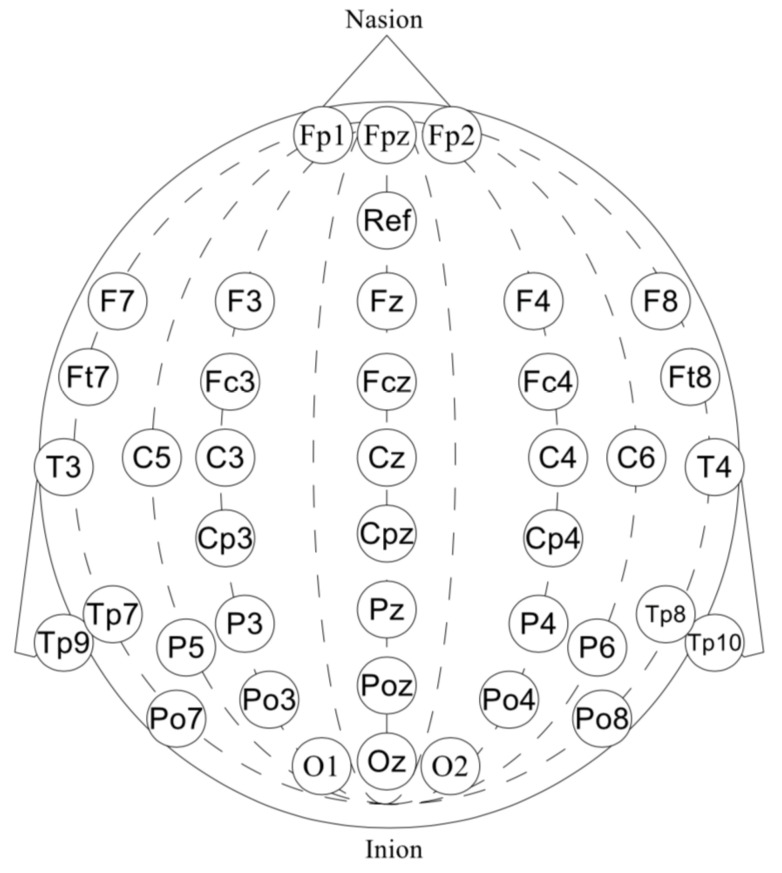
The 40-channel EEG: ‘Fp1’, ‘Fpz’, ‘Fp2’, ‘F7’, ‘F3’, ‘Fz’, ‘F4’, ‘F8’, ‘Ft7’, ‘Fc3’, ‘Fcz’, ‘Fc4’, ‘Ft8’, ‘T3’, ‘C3’, ‘Cz’, ‘C4’, ‘T4’, ‘Tp7’, ‘Cp3’, ‘Cpz’, ‘Cp4’, ‘Tp8’, ‘C5’, ‘Tp9’, ‘P3’, ‘Pz’, ‘P4’, ‘Tp10’, ‘C6’, ‘P5’, ‘Po3’, ‘Poz’, ‘Po4’, ‘P6’, ‘Po7’, ‘O1’, ‘Oz’, ‘O2’, ‘Po8’.

Our dataset consists of 40-channel EEG signal recorded at 500 Hz. EEG was acquired by “wet” Ag/AgCl electrodes with a NVX52 bio-signal amplifier and NeoRec recording software in a BDF+ file format [33,34]. The electrodes were positioned according to the standard 10–20 scheme with the additional electrodes in the areas of interest. For the temporal areas, see Figure 2. The ‘Afz’-channel was used as a reference electrode. A word presentation signal was also captured via a light sensor and included in data files as a mark.

**Figure 3:**
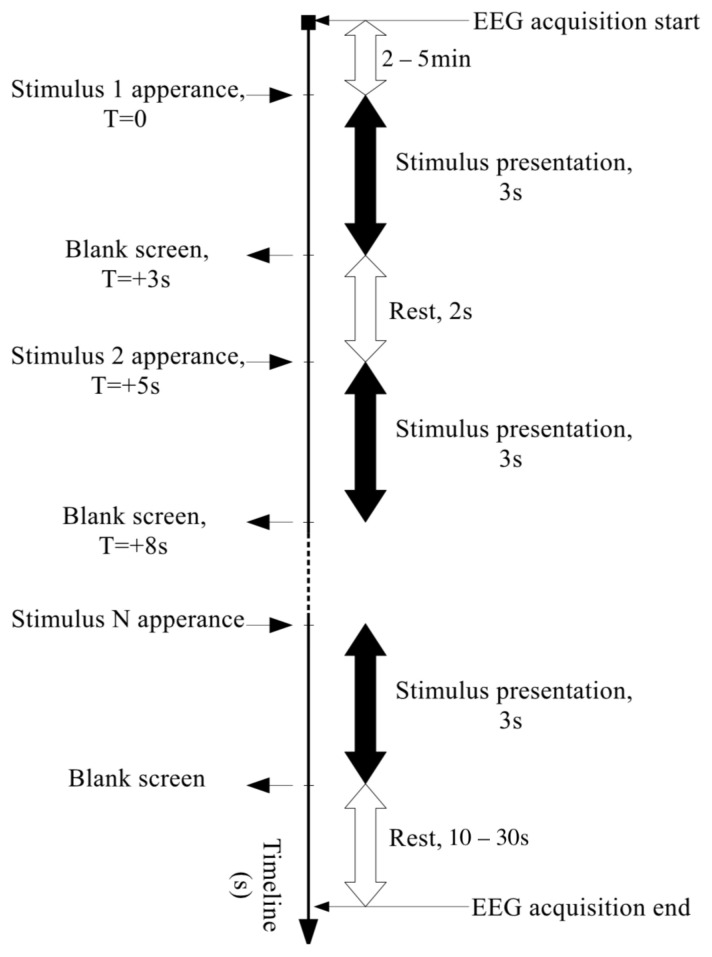
Experiment time scheme.

Each experiment lasted from 5 to 14 sessions, depending on the participant’s condition and preference. Each session consisted of reading aloud 10 random words from the screen (vocalized speech) or silently (silent speech). During the session, each word was displayed on the screen for 3 s, then the screen turned off for 2 s (Figure 3).

### 2.2. Eye Noise Filtering

We developed a three-step algorithm to filter eye noise (blinking and eye movement). For our study, we decided to consider the eye noise independent of brain activity.

**Figure 4:**
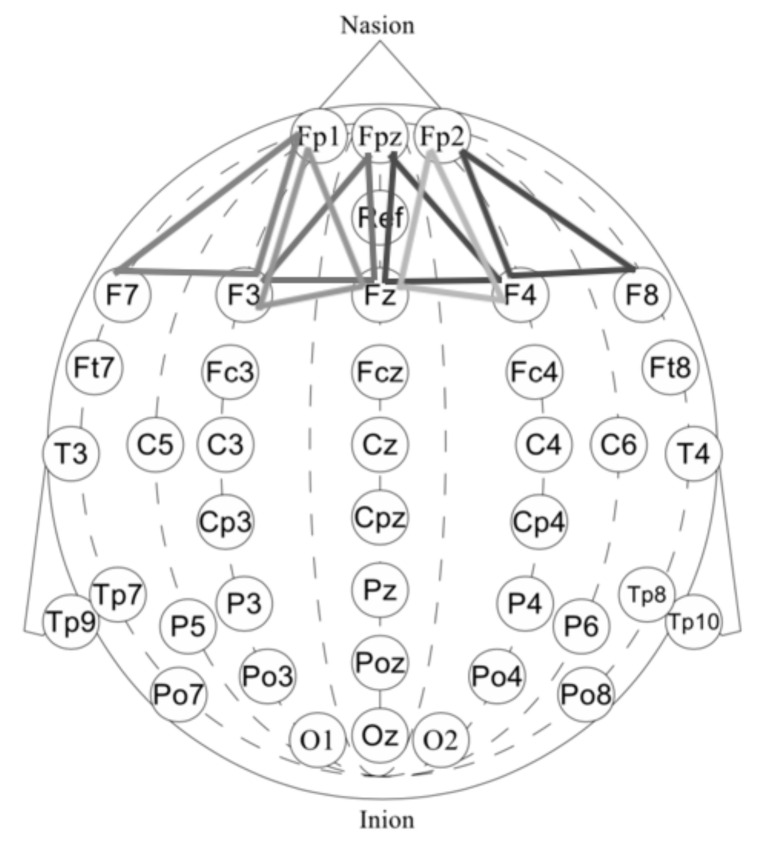
Frontal lobe electrode grouping. Frontal channels (electrodes) were grouped into four sets of three electrodes, taking into account the proximity and location to one side of the reference electrode: ‘Fpz’, ‘Fz’, ‘F3’, ‘Fp1’, ‘F3’, ‘F7’, ‘Fpz, ‘Fz’, ‘F4’, ‘Fp2’, ‘F4’, ‘F8’.

**Figure 5:**
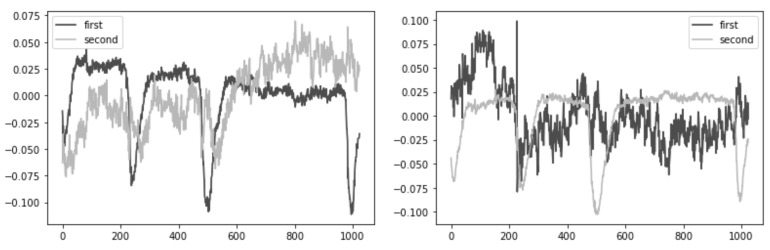
Plots A and B show that the eye noise component is randomly determined sometimes as the first component and sometimes as the second component.

- First, we performed a morphological selection of electrodes. To identify eye noise, we chose the most eye-sensitive electrodes located in the frontal region. We grouped the frontal electrodes into four sets of three electrodes, taking into account the proximity and location to one side of the reference electrode: {‘Fpz’, ‘Fz’, ‘F3’}, {‘Fp1’, ‘F3’, ‘F7’}, {‘Fpz’, ‘Fz’, ‘F4’}, {‘Fp2’, ‘F4’, ‘F8’}. See Figure 4 for the location of the electrode groups on the head.
- Second, we performed an Independent Component Analysis (ICA). We used ICA on the grouped frontal electrodes to decompose a multivariate signal into independent non-Gaussian signals: eye noise and brain activity during silent speech. For each triad of the frontal electrodes ({‘Fpz’, ‘Fz’, ‘F3’}, {‘Fp1’, ‘F3’, ‘F7’}, {‘Fpz’, ‘Fz’, ‘F4’}, {‘Fp2’, ‘F4’, ‘F8’}) ICA was applied to get two components. We used the sklearn library to apply ICA to our data with the following parameters: sklearn.decomposition.FastICA (n_components=2,algorithm=deflation,random_state=42). However, as you can see in the picture, the components were not always selected in the correct order (Figure 5). To select the correct component, we applied the following method.
- Third, we correctly identified and isolated the eye noise component. Separating the eye noise component required three steps as well. During the first step, we performed a Fast Fourier transform (FFT) for each component and cut the lowest 3 frequencies to remove a trend, and right after we performed an inverse Fourier transform. During the second step, the resulting two components were smoothed using the Savitsky–Galey filter (savgolfilterfromscipy.signal) with the following parameters: savgolfilter(component,21,k=5). During the third step, we calculated the median and maximum value for each component. Finally, we selected the component with the largest difference between the median and maximum values and considered this the eye noise component.

Note Bene, this procedure does not cause loss of signal information, since the whole procedure is aimed at finding the low-frequency component corresponding to eye blinking.

### 2.3. Dimensions Reduction and Component Analysis

First, we began by selecting the EEG-data obtained from the electrodes for the left temporal group (‘T3’, ‘Ft7’, ‘C7’, ‘Tp7’) due to its proximity to the speech center. To filter out tensors with a high noise level, as well as tensors with missing data, two filters were applied by the sum of the moduli of the signal amplitudes. The cutoff value for the second filter was chosen as the 95th percentile of the total moduli of the amplitudes of the remaining tensors. After filtering, the data was converted into the frequencies using the Fourier transform; frequencies below 5 Hz and above 49 Hz were brought to zero. The resulting data obtained were sequentially reduced to 50 components by the principal component analysis (PCA) method and right after down to 3 components by the t-SNE method (perplexity=30, learning_rate=10, n_iter = 5000).

### 2.4. Kolmogorov–Smirnov Test

The resulting distributions of the three components were grouped relative to the words pronounced by the subjects and analyzed for belonging to the same general population by the Kolmogorov–Smirnov method. Then we identified statistically significant differences in pairs for all words.

### 2.5. Downsampling

**Figure 6:**
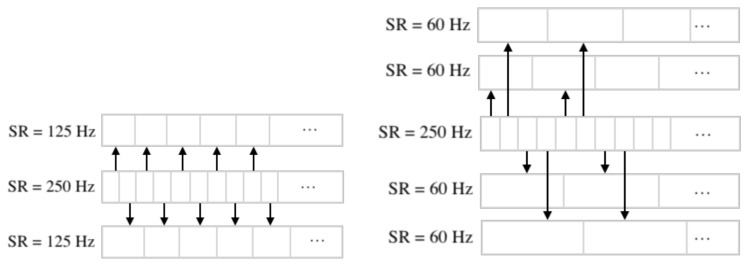
Downsampling from 250 Hz to 125 Hz and downsampling from 250 Hz to 62.5 Hz.

The sampling rate was downsampled using index masks on the original EEG-data. For example, when we perform downsampling from 250 Hz to 125 Hz, the first array contains elements with even indices, while the second contains elements with odd ones. In the case of lowering the frequency to 62.5 Hz, the indices are selected according to the remainder when divided by 4. A more visual and detailed downsampling method is shown in Figure 6.

### 2.6. Separation of Electrodes into Left and Right Hemispheres

**Figure 7:**
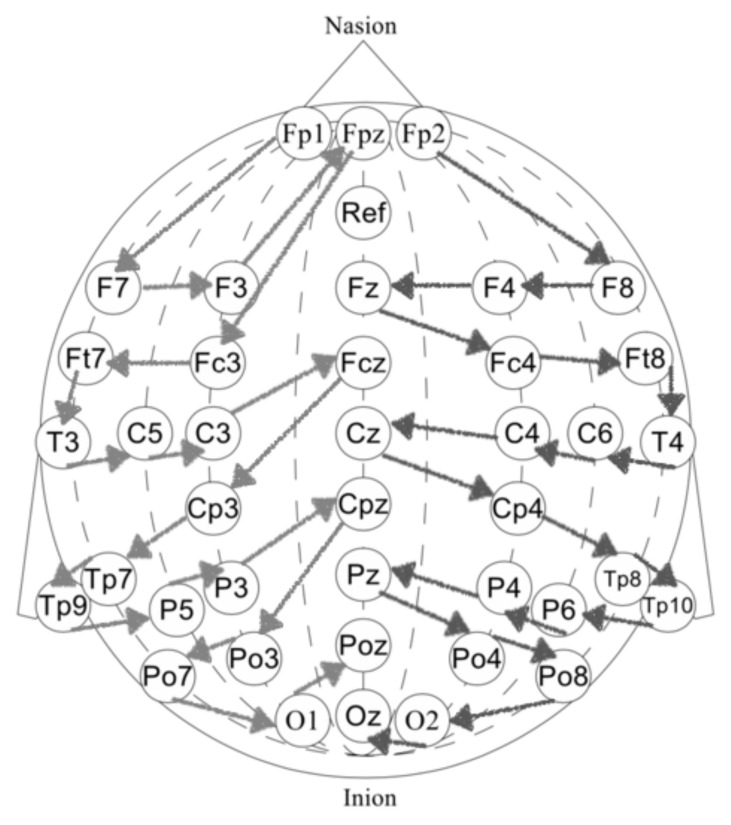
Separation of electrodes into the left and right hemispheres with number of channels and spatial balance between hemispheres.

Separation of electrodes into left and right hemispheres was performed taking into account the spatial balance between hemispheres and can be viewed in Figure 7.

### 2.7. Presenting the EEG-Data as a Two-Dimensional Vector

First, we cut the EEG-data into 2D vectors—2048 long (which is about 4 s, considering the sampling rate is equal to 500 Hz) and 40 wide (which is the number of EEG channels).

Second, using the downsampling algorithms presented above, the sampling rate was downsampled from 500 Hz to 250 Hz, and the data were split into two separate samples (let us call them ‘Sample 01’ and ‘Sample 02’) with dimensions 40×1024.

Third, using the downsampling algorithms presented above, we downsampled Sample 01 (Sample 02 separately) from 250 Hz to 125 Hz and packed the resulting samples into a 2D vector (80×512) and cut the first half into the 2D vector (80×256) each. In parallel, the sampling rate is downsampled from 125 Hz to 62.5 Hz in parallel for Sample 01 (Sample 02 separately) and the resulting samples are packed into a 2D vector (160×256).

Fourth, in parallel, using the Eye Noise Filtering presented above, we obtained 6 components of eye noise (6×256) from 6 groups of channels from Sample 01 (Sample 02).

Fifth, taking into account the separation of electrodes into left and right hemispheres we combined vectors into the 2D vector (256×256). All obtained vectors were combined according to the following order: eye noise of the left hemisphere, downsampled tensors from the left hemisphere, median from ‘Ft7’ and ‘T3’ channels, downsampled tensors from the left and right hemispheres respectively, median from ‘Ft8’ and ‘T4’ channels, downsampled tensors from the right hemisphere, eye noise of the right hemisphere.

**Figure 8:**
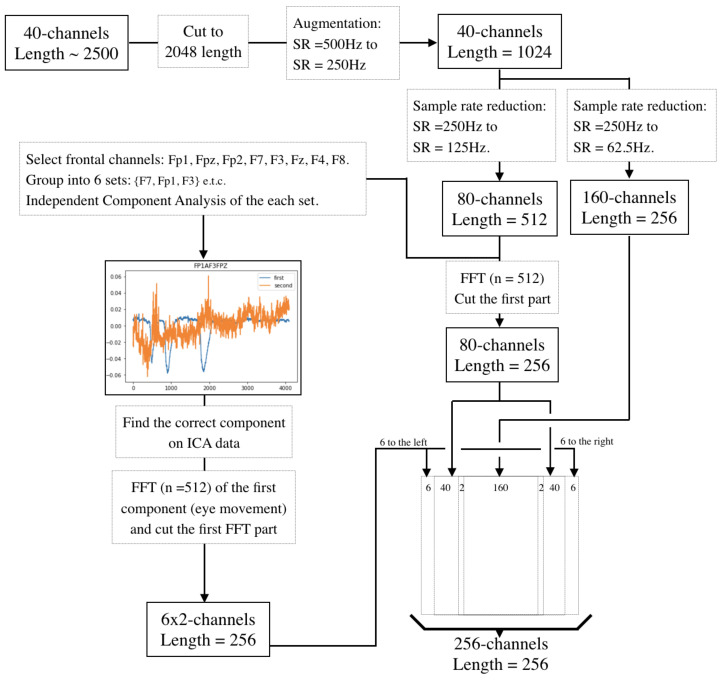
Presenting EEG-data as two-dimensional vectors.

The complete preprocessing scheme can be seen in Figure 8.

The use of such downsampling made it possible to represent the data in the form of a square containing fairly evenly distributed information about the signal without any information losses. Thus, the resulting 256×256 tensor contains preprocessed data, additional features, and data with minimal processing, which in steps to come can help the neural network to identify patterns of silent speech. The preliminary data processing takes into account not only the presence of noise but also the peculiarities of their location.

### 2.8. Neural Networks

**Figure 9:**
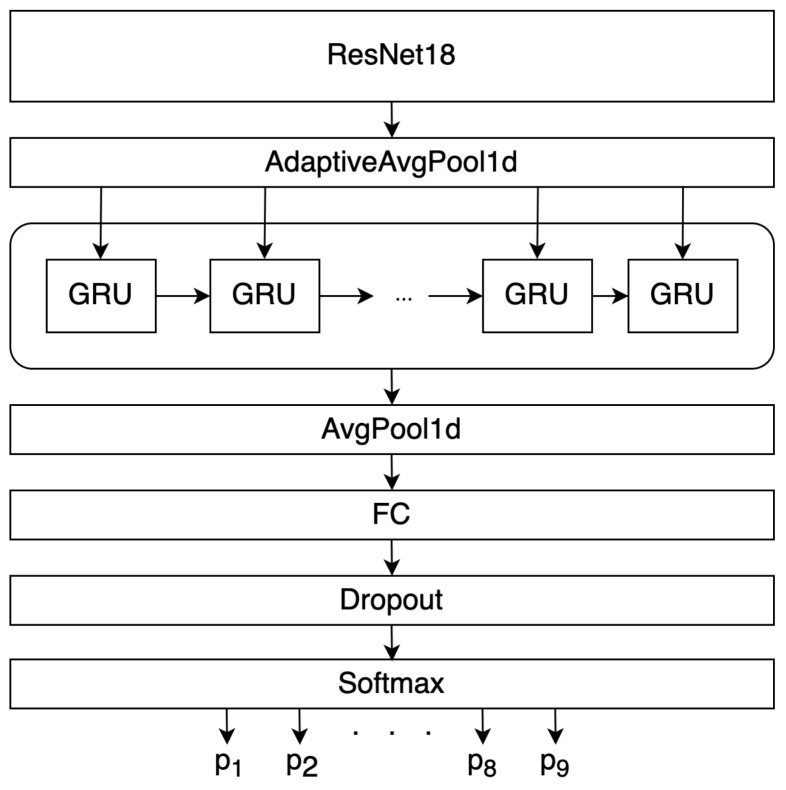
“ResNet18 + 2GRU” network architecture.

First, as a classifier, we used a model that includes the convolutional neural networks ResNet18/50/101 with 2 layers of controlled recurrent units—Gated Recurrent Unit (GRU) [35]. At the beginning of ResNet, convolution, BatchNorm2D, and the ReLU activation function were added, in order to adapt to the preprocessed data. This architecture does not use the last fully connected layer nor the Softmax function used in the original architecture of this network. To further reveal the patterns, the features obtained as a result of the ResNet18 operation are fed to the input of a recurrent neural network containing 1024 hidden GRUs. A similar approach is used in problems of image description [36]. Into the recurrent neural network we used an average one-dimensional pooling with a filter size of 2, as a result of which 512 values were transmitted to the input of a fully connected layer. ReLU was used as the activation function for a fully connected layer [37]. To normalize the Neural Network outputs, we used the Softmax function. Our “ResNet18 + 2GRU” network architecture can be seen in Figure 9. We did not use dropout. We used Adam with a mini-batch size of 16. The learning rate starts from 0.01 and is divided by 10 two times every 500th epoch. The models were trained for up to 2500 iterations. Each sample (from 1, 2, ..., 256 individuals) before training was divided into training (70%), validation (20%), and test (10%), we also used out-of-sample test sample. The validation set was not used to adjust the weights of the networks.

In order to test hypothesis 2 about a different way of reproducing silent speech by each person, the presented architectures were trained on the data of each person separately. Further, we doubled the number of individuals, reaching the entire dataset for training.

## 3. Results

### 3.1. Dimensions Reduction, Component Analysis and Kolmogorov–Smirnov Test

**Figure 10:**
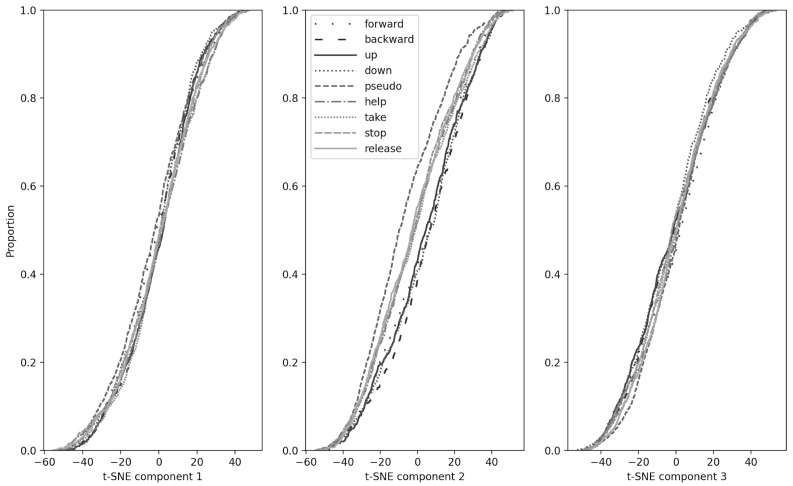
The resulting distributions of the three components of t-SNE were grouped relative to the words silently “pronounced” by the subjects and analyzed for belonging to the same general population by the Kolmogorov–Smirnov method.

We applied the PCA method and the t-SNE method to the EEG-data sequentially and obtained three components for each word. Then, the resulting three component vectors were grouped relative to the words pronounced by the subjects. Each such group was viewed as a separate sample, distributed in a certain way. The pairwise Kolmogorov–Smirnov test for checking if samples (represented by different words) belong to the same general sample showed that the distributions of all words in pairs are statistically significantly distinguishable. We obtained that EEG-data from all different words belong to different distributions. These results can be seen in more detail in Figure 10 and Table 3. We obtained data showing that the distributions of movement commands have similar statistics and overlap strongly. Moreover, as expected, the pseudoword distribution is much more statistically separate from the rest of the distributions. On out-of-sample data, the accuracy exceeded random, but was very modest.

We have shown that word distributions are statistically distinct. The results obtained showed us the fundamental validity of further classification using neural networks.

### 3.2. Neural Networks Classification

We tested the work of the classifiers on different neural networks. Specifically, we have tried: “ResNet18 + 2GRU”, “ResNet50 + 2GRU”, “ResNet101 + 2GRU”, and “Inception ResNet V2”. The best results were obtained on a “ResNet18 + 2GRU” neural network. The results of the “ResNet18 + 2GRU” neural network operation are shown in Table 4. The maximum accuracy in the classification of 9 words was achieved when training on a limited group of people, where the test sample was selected from the data of this group (training was not performed on the test sample). The accuracy on the test under these conditions reached 84.51%. With binary classification, the accuracy under such training conditions reached 90.43%.

The accuracy of the neural network binary classifier when tested on out-of-sample data was better than random prediction and reached 53%.

## 4. Discussion

In conducting this research, we paid attention to both practical as well as fundamental questions, the answers to which would show us what we need to efficiently build BCI communication. As concerns practical matters, we have shown once again that recognition of mental commands is indeed possible through the construction of non-invasive BCIs based on EEG. In addition, we formulated and confirmed three hypotheses about individual development and patterns of brain activity.

Regarding our first hypothesis, individual brain development is characterized by population-specific patterns of brain electrical activity during silent speech. Humans have some common brain responses to words during silent speech. This statement is substantiated by the fact that the EEG-recorded silent speech of 9 words from different people form distributions corresponding to each word; these distributions are statistically separable (using the Kolmogorov–Smirnov method) and separable by the neural network classifier (“ResNet18 + 2GRU”). The presence of such nonlinear separable distributions indicates the presence of common brain activity patterns in the group of subjects. These common patterns were not obvious and were distinguished by a small set of features; however, it was possible to recognize silent words by the electrical activity of the brain, even in people on whom we did not train the neural network. Our data showed that the learning outcomes on a limited sample are portable to the general population. Indeed, the accuracy of the binary classifier when tested on out-of-sample data was not impressive (52%) on average, but it was better than random prediction. However, it makes sense to train the neural network on a large dataset of silent speech, which will allow us to expect an increase in the accuracy of recognition of silent speech on out-of-sample data.

Regarding our second hypothesis, we have confirmed that the differences between people are so strong that additional BCI training on one person is more effective for the specific person than expanding the dataset on other people. By training on one person (or a small group of people), and testing the results of the neural network on the data recorded from this person (or group), we got impressive accuracy during silent speech, or as we call it, “mental commands”. Indeed, we have shown that the classification accuracy for training on one individual reached 84.5% for 9 words and 87.9% for binary classification. At the same time, a network trained on one individual showed a random result (almost the same as chance) when recognizing words on a sample of people on whom the network was not trained. That is, the brain of one person, when pronouncing one word with an inner voice (silent speech), does it quite conservatively. Individual conservatism is much more pronounced than population conservatism. Such a difference in accuracy when training on one individual and on a group of people can be explained by a large difference in ontogenesis. However, we have assumed (we already have preliminary results about occupational clustering) that there are groups of people who have ontogenetic similarities. Our next publication will be devoted to this problem.

Once again, the individual brain has brain activation patterns for mental commands, which allows us to train a neural network classifier on the data obtained from one individual with excellent accuracy. What is especially important, is that for such a neural network classifier we do not even need to give the subject feedback—whether she or he has “silently pronounced” the word correctly or not. The presence of such individual patterns of brain activity allows us to implement neural network to BCIs without adapting individuals themself to a BCI neural network classifier (without feedback). The lack of this need for additional user training is quite remarkable, since most BCIs require it training.

Regarding the question of how strongly the words are similar with respect to the activity they elicit in the brain during silent speech, we have shown that different silent words give different distributions. Semantically similar words describing directed movements (‘up’, ‘down’, ‘forward’, ‘back’) give similar distributions, and are predictably poorly classified by the neural network classifier. It is interesting that in Russia these movement words (‘up’, ‘down’, ‘forward’, ‘back’) have different articulations and different phonetics, but some semantic similarity compared to more distant words such as ‘take’, ‘release’, and ‘help’. At the same time, we see that despite the phonetic and articulatory difference, these words (‘up’, ‘down’, ‘forward’, ‘back’) have similar distributions, and, as expected, are classified worse by the neural network. Thus, even in our modest vocabulary, we can say there are words that trigger similar patterns of brain activity during silent speech. Moreover, phonetic proximity of words gives much less similarity than semantic proximity.

## 5. Conclusions

In this work, we investigated silent speech using EEG-data from the point of view of developing BCIs for people with disabilities. We achieved 84.5% accuracy performing 9 words (including a pseudoword) classification and 88% accuracy in binary classification on average. This is such an accuracy of silent speech recognition expressed by 9 thinking commands, which is comparable to the best results of our colleagues. Based on these results, we can conclude that our preprocessing and neural network classifier are applicable for implementation in EEG-based BCI.

Using the Kolmogorov–Smirnov method, we showed the nonlinear separability of the distributions represented by the EEG signal recorded during the silent speech. We have shown that these distributions for different words are nonlinearly separable by statistical methods and methods using neural networks.

We got some insights into the patterns of electrical activity in relation to human brain development and the statistical distribution of silent words. We also formulated and confirmed three hypotheses. First, we confirmed that individual development of the brain has electrical activity patterns constant across the human population when solving silent speech problems. It means that training a neural network on EEG-data from a limited group of people can be used to develop EEG-based BCI for other people. This is especially important when we are talking about creating a universal device that can meet the needs of people of different groups. In particular, the fact that learning can be transferred to people with disabilities without tormenting them with collecting data.

Second, we confirmed that a smaller dataset collected on one subject made it possible to build a more accurate neural network classifier for a given subject than a larger dataset collected on a group of people. Thus, individual ontogenesis of the brain has individual patterns of electrical activity of the brain in solving silent speech problems.

Third, we confirmed that silent words distributions can be very close and there are words that share similar patterns of brain activity. However, the commands that we have chosen for our dataset, as we have shown, can be separated by a neural network classifier in EEG-based BCI. This fact is important for our further work because it allows developers to build BCI for paralyzed people.

## Figures and Tables

**Table 1:** Comparison of existing works focused on silent speech recognition using biological electrical activity.

Algorithm	Data Type	Accuracy on NN Set Size				Year
		Binary	3	4	> 4	
Spatial filters and support vector machines [7]	Phonemes: /a/, /u/	78%	-	-	-	2009
K-nearest neighbor algorithm and autoregression [8]	Syllables: /ba/, /ku/	61%	-	-	-	2010
Extreme learning machine with radial basis function and linear discriminant analysis [9]	Phonemes: /a/, /e/, /i/, /o/, /u/.	72%	-	-	-	2016
Support vector machine on the Riemannian space [12]	Phonemes: /a/, /i/, /u/	49.2%	-	-	-	2017
	Words: ’in’, ’cooperate’	80.1%	-	-	-	
Support vector machine and neural network [11]	English words: ‘yes’, ‘no’. Hindi words: ‘haan’, ‘na’.	75.4%	-	-	-	2017
Wavelet transform and regularized neural network [21]	Words: ‘yes’, ‘no’.	67%	-	-	-	2017
Support vector machine [13]	Syllables: /iy/, /piy/, /tiy/, /diy/, /uw/, /m/, /n/. Words: ‘pat’, ‘pot’, ‘knew’, ‘gnaw’.	-	-	33.33%	-	2018
Three-layer convolutional neural network on myography data [22]	Numbers 0 to 9 in English.	-	-	-	92.01%	2018
Hierarchical conventional neural network, deep autoencoder and extreme gradient boosting [15]	Syllables: /iy/, /piy/, /tiy/, /diy/, /uw/, /m/, /n/. Words: ‘pat’, ‘pot’, ‘knew’, ‘gnaw’.	83.42%	-	-	66.2%	2019
Recurrent network [25]	Phonemes: /a/, /e/, /i/, /o/, /u/.	-	-	-	98.61%	2019
	Words: ‘yes’, ‘no’, ‘left’, ‘right’.	-	-	99.38%	-	
Recurrent network [24]	Words with a consonant-vowel-consonant structure.	72%	-	-	-	2019
Kernel-based extreme learning machine [14]	Words: ‘left’, ‘right’, ‘up’, ‘down’	85.57%	-	49.77%	-	2020
Five-layer convolutional neural network with the final fully connected layer [26]	15 English sentences from 3 participants.	-	-	-	81%	2020
Time and space convolution networks [27]	Phonemes: /a/, /e/, /i/, /o/, /u/.	-	-	-	30%	2020
Hierarchical neural network consisting of two convolutional neural networks and the k-nearest neighbors algorithm [28]	Spanish words: ‘arriba’, ‘abajo’, ‘derecha’, ‘izquierda’, ‘adelante’, ’atrás’.	-	-	24.97%	31.40 ± 2.73%	2020

**Table 2:** Subjects, grouped by age, education, sex, and professional area of current work.

Sex	Professional	Sample	Mean	Std	Min	Max
	Area	Number	Age		Age	Age
Female	Formal and natural sciences	27	23.8	5.5	18	38
Female	Humanities, social and applied science	72	28.0	8.8	18	56
Female	Medicine and biology	63	22.1	5.4	18	48
Male	Formal and natural sciences	39	27.6	8.1	18	46
Male	Humanities, social and applied science	34	28.1	7.6	18	47
Male	Medicine and biology	33	22.8	6.7	18	48

**Table 3:** Kolmogorov–Smirnov test. Statistics and *p*-value (statistically significant differences) for the distributions of EEG-data corresponding to different words in silent speech.

Word:	‘backward’	‘up’	‘down’	pseudo	‘help’	‘take’	‘stop’	‘release’
‘forward’								
Component ${}^{*}$	3	1	1	2	2	2	2	2
Statistics	0.046	0.047	0.023	0.117	0.161	0.125	0.175	0.137
*p*-value	0.0109	0.009	0.0027	$3.54\times 10^{-14}$	$1.86\times 10^{-23}$	$1.54\times 10^{-14}$	$1.29\times 10^{-14}$	$3.54\times 10^{-14}$
‘backward’								
Component ${}^{*}$		1	3	2	2	2	2	2
Statistics		0.056	0.059	0.059	0.167	0.13	0.155	0.14
*p*-value		0.0006	0.0003	$2.54\times 10^{-14}$	$1.02\times 10^{-26}$	$4.18\times 10^{-17}$	$9.26\times 10^{-25}$	$2.6\times 10^{-14}$
‘up’								
Component ${}^{*}$			2	2	2	2	2	2
Statistics			0.0269	0.14	0.142	0.104	0.144	0.12
*p*-value			0.00015	$9.11\times 10^{-21}$	$2.19\times 10^{-19}$	$3.89\times 10^{-11}$	$2.54\times 10^{-14}$	$9.1\times 10^{-14}$
‘down’								
Component ${}^{*}$				2	2	2	2	2
Statistics				0.128	0.147	0.114	0.156	0.126
*p*-value				$9.24\times 10^{-17}$	$6.18\times 10^{-20}$	$7.31\times 10^{-13}$	$2\times 10^{-15}$	$3.54\times 10^{-14}$
pseudo								
Component ${}^{*}$					2	2	2	2
Statistics					0.242	0.233	0.27	0.23
*p*-value					$2.54\times 10^{-14}$	$1.47\times 10^{-51}$	$3.11\times 10^{-71}$	$1.27\times 10^{-46}$
‘help’								
Component ${}^{*}$						3	1	3
Statistics						0.063	0.052	0.095
*p*-value						0.001	0.0096	$2.27\times 10^{-7}$
‘take’								
Component ${}^{*}$							2	3
Statistics							0.054	0.074
*p*-value							0.0057	$8.48\times 10^{-5}$
‘stop’								
Component ${}^{*}$								3
Statistics								0.074
*p*-value								$7.67\times 10^{-5}$

${}^{*}$—Number of the component where the statistical test is most convincing.

**Table 4:** Results of “ResNet18 + 2GRU”.

	9 Silent Words			2 Silent Words		
	Limited		Out-of-Sample	Limited		Out-of-Sample
	Data		Data	Data		Data
Training	Train	Test	Test	Train	Test	Test
Sample	Accuracy/	Accuracy/	Accuracy/	Accuracy/	Accuracy/	Accuracy/
Cardinality	Loss	Loss	Loss	Loss	Loss	Loss
1	91.98%/	84.51%/	10.79%/	100%/	87.92%/	52%/
	1.52	1.46	2.26	0.46	0.37	0.74
2	80.52%/	82.34%/	10.1%/	100%/	88.21%/	52.3%/
	1.56	1.55	2.26	0.39	0.31	0.77
32	74.28%/	18.91%/	13.15%/	70%/	89.5%/	51.4%/
	1.91	2.18	2.23	0.43	0.69	0.84
256	72.34%/	13.39%/	10.21%/	66.04%/	85.18%/	52.3%/
	1.64	2.16	2.2	0.41	0.62	0.77

## Abbreviations

The following abbreviations are used in this manuscript:

MDPI	Multidisciplinary Digital Publishing Institute
PJSC	Public Joint-Stock Company
ML	Machine Learning
EEG	Electroencephalography
BCI	Brain–computer Interface
ICA	Independent Component Analysis
t-SNE	t-distributed Stochastic Neighbor Embedding
PCA	Principal Component Analysis
CNN	Convolutional Neural Network
FFT	Fast Fourier Transform
GRU	Gated Recurrent Unit
SVM	Support Vector Machine
